# A remarkable new genus of stiletto flies from Egypt, with a key to Palaearctic genera of Phycinae (Diptera, Therevidae)

**DOI:** 10.3897/zookeys.184.2759

**Published:** 2012-04-21

**Authors:** Shaun L. Winterton, Martin Hauser, Haitham B.M. Badrawy

**Affiliations:** 1 California State Collection of Arthropods, California Department of Food & Agriculture, Sacramento, California, USA; 2 Entomology Department, Ain Shams University, Cairo, Egypt

**Keywords:** Asiloidea, Therevidae, Phycinae

## Abstract

An unusual new genus (*Salwaea burgensis* **gen. n., sp. n.**) of phycine stiletto flies is described from Egypt. A key to Palaearctic genera of Phycinae is presented.

## Introduction

The stiletto fly subfamily Phycinae comprises 128 species in 18 genera (four extinct) distributed in all major biogeographic regions except Australasia (Hauser 2005; Hauser and Webb 2007). Diagnostic characters used to differentiate members of this subfamily include absence of lanceolate setae on the femora, setulae on wing vein R_1_ (although absent in *Schlingeria* Irwin, 1977), wing costal vein terminating before vein CuA_2_ (never circumambient), palpi with an apical pit, female terminalia with only a single set of variously developed A1 spines on the acanthophorite (tergite 10), abdominal tergites 9 and 10 as separate sclerites, three spermathecae and absence of a spermathecal sac (Lyneborg 1972; Hauser 2005).

The nominal genus, *Phycus* Walker, 1850, is not only the most species-rich genus of Phycinae (25 spp.), it is also the most widely distributed with species recorded throughout the Oriental, Palaearctic, Afrotropical, Nearctic and Neotropical regions (Lyneborg 2003). Five extant genera occur in the New World, *Ataenogera* Kröber, 1914 (6 spp.), *Parapherocera* Irwin, 1977 (3 spp.), *Pherocera* Cole, 1923 (11 spp.), *Schlingeria* Irwin, 1977 (1 sp.) and *Phycus* Walker, 1850 (Irwin 1977, 1983; Hauser 2005; Hauser and Webb 2007). Phycinae are more diverse and species-rich in the Palaearctic and Afrotropical regions with many genera found in both regions, including *Actorthia* Kröber, 1912 (13 spp.), *Phycus* Walker, 1850, *Acathrito* Lyneborg, 1983 (9 spp.) and *Ruppellia* Wiedemann, 1830 (5 spp.) (Lyneborg 1983, 1989, 2003; Badrawy and Mohammad 2011). *Neotabuda* Kröber, 1931 (20 spp.), *Orthactia* Kröber, 1912 (7 spp.) and *Stenogephyra* Lyneborg, 1987 (7 spp.) are restricted to the Afrotropical region, being largely endemic to southern Africa (Lyneborg 1980, 1988; Hauser 2005; Webb and Hauser 2011). In contrast, three genera are restricted to the Palaearctic region; *Efflatouniella* Kröber, 1927 (3 spp.) and *Yemenia* Koçak & Kemal, 2009 (1 sp.) are found in the Middle East while *Salentia* Costa, 1857 (11 spp.) is more widely distributed (Lyneborg 1983; Hauser 2005; Mohammad and Badrawy 2011). Four fossil genera of Phycinae are known: *Kroeberiella* Hauser, 2007, *Dasystethos* Hauser, 2007, *Glaesorthactia* Hennig, 1967 (all from Baltic Amber) and *Palaeopherocera* Hauser, 2005 (Florissant) (Hauser 2005, 2007).

Seven phycine genera are currently known from Egypt and the Middle East (*Salentia*, *Phycus*, *Actorthia*, *Acathrito*, *Ruppellia*, *Efflatouniella* and *Yemenia*) (Hauser 2005; Mohammad and Badrawy 2011; Badrawy and Mohammad 2011). Recent examinations of material collected during the early part of the 20^th^ century by the renowned Egyptian entomologist Hassan C. Efflatoun, has uncovered an unusual new therevid from northern Egypt. This species represents a remarkable new genus of Phycinae with particular characteristics not seen previously in Therevidae. Although only a single female specimen is known, *Salwaea burgensis* gen. *et* sp. n. is diagnosed from other phycine genera by distinctively shaped head and antennae and termination of the costal vein in the radial field. This new genus and species is described and figured herein, and a dichotomous key to Palaearctic genera of Phycinae is presented.

## Materials and methods

Adult morphological terminology follows McAlpine (1981) and Hauser (2005) with genitalic morphology as modified by Winterton et al. (1999a,b) and Winterton (2006). Genitalia were macerated in 10% KOH to remove soft tissue, then rinsed in distilled water and dilute glacial acetic acid, and dissected in 80% ethanol. Genitalia preparations were placed in glycerine in a genitalia vial mounted on the pin beneath the specimen.

Specimen images were taken at different focal points using a digital camera and subsequently combined into a serial montage image using Helicon Focus software. All new nomenclatural acts and literature are registered in ZooBank (Pyle and Michel 2008).

## Taxonomy

### Salwaea gen. n.

urn:lsid:zoobank.org:act:D47A75EA-5ACF-414E-AE67-BC9C5239C28E

http://species-id.net/wiki/Salwaea

#### Type species.

*Salwaea burgensis* sp. n. (by present designation)

#### Diagnosis. 

Body length: 6.5 mm (female). Antenna longer than head; enlarged bulbous scape with erect strong macrosetae (absent medially); pedicel medio-dorsally inserted on scape; flagellum two-segmented, large paddle-like first flagellomere longer than combined scape and pedicel length, style apical; parafacial without setae; proboscis barely protruding from oral cavity; prosternal depression and mid coxa without setae; metanepisternum without postspiracular setae; hind coxal knob present; costal vein ending just beyond R_4_; R_1_ with single row of setulae; M_1_ and M_2_ terminating before wing margin; costal margin with scattered setae, not arranged in two rows; cell m_3_ closed, petiolate to margin; distal tarsomere with pulvilli and claws relatively small, aligned with axis of leg, dorsal seta[e] on distal tarsomere elongate and projecting; sternite 8 rounded, strongly convex, posteriorly emarginate; tergite 10 as paired sclerites, not fused medially; acanthophorite spines greatly reduced in size.

#### Etymology.

This genus is named in honour of Prof. Dr. Salwa K. Mohammad. Gender is feminine.

#### Relationship to other phycine genera.

It is difficult to identify characters likely to support the phylogenetic placement of *Salwaea burgensis* gen. *et* sp. n., as it exhibits a series of both autapomorphies, unique to the taxon, and plesiomorphies, which it shares with all genera of Phycinae. Notable characters found only in this new genus are the large paddle-like shape of the flagellum and the termination of the costal vein just beyond where R_4_ joins the wing margin. In all other genera the flagellum ranges from cylindrical to turbinate, while the costal vein is either circumambient (most Therevinae and Agapophytinae), terminates at M_2_ (Xestomyzinae) or terminates at various points typically between M_1_ and CuA_2_ (Phycinae). In rare cases in all subfamilies except Xestomyzinae, individual species have the costal vein terminating in the radial field (typically R_5_) with medial veins terminating before the wing margin. The reduction of the pulvilli is shared with some species of *Pherocera*, *Orthactia* and *Phycus*. In the latter genus the following species have completely reduced pulvilli: *Phycus annulipes*, Lyneborg 1978 (Kenya), *Phycus flavus* Lyneborg, 1978 (South Africa), *Phycus lacteipennis* Lyneborg, 2002 (Morocco), *Phycus marginatus* Kröber, 1912 (Egypt, Chad, Congo, Mali, Nigeria, Senegal, Sudan) and *Phycus mirabilis* Lyneborg, 1978 (Botswana, South Africa, Zimbabwe). Some *Phycus* species have very short and barely discernable pulvilli that may consist only of a pulvillus sclerite remnant: *Phycus angustifrons* Lyneborg, 2003 (Thailand), *Phycus insignis* Loew, 1874 (Egypt, Middle East to Central Asia), *Phycus kroeberi* (Brauns, 1924) (South Africa), and *Phycus stylatus* Lyneborg, 1978 (Kenya, Botswana, Tanzania). Although there is no resolved species-level phylogeny for the genus *Phycus*, taxonomic affinities suggest that pulvilli reduction occurred several times independently in this genus as a derived feature.According to Kröber (1927) the pulvilli are absent in *Efflatouniella*, although specimens examined have pulvilli that are slightly reduced in a few specimens. The enlarged scape is also found in *Neotabuda* and *Salentia*, putative sister genera according to Lyneborg (1980) and Hauser (2005). The furca of *Salwaea* gen. n. has a distinctive quadrangular shape, suggesting a close relationship with *Salentia* or *Phycus* or, perhaps, the pleisomorphic condition of the furca in Phycinae (Fig. 5). *Actorthia*, *Acathrito* and *Efflatouniella* display a very different configuration of the furca whereas the furca of two species of *Neotabuda* examined are unique among phycines. The furca of *Salwaea* gen. n.is typical for Phycinae, which always has two compartments, in contrast to the undivided furca type of higher Therevidae like *Thereva*. The presence of an anteroventral seta on the fore and hind femora in *Salwaea* gen. n. is also found in *Salentia* and *Neotabuda*, but not with *Phycus*.

### Salwaea burgensis sp. n.

urn:lsid:zoobank.org:act:3F23A487-AFD0-4734-BBBC-09E4E1B67DB4

http://species-id.net/wiki/Salwaea_burgensis

**Figure 1.**
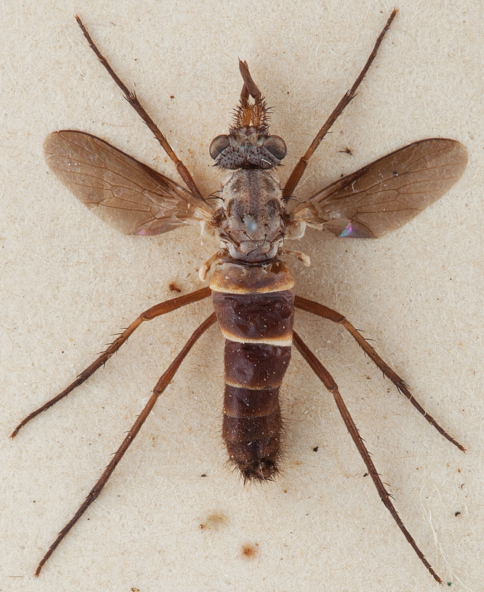
*Salwaea burgensis* gen. et sp. n., female Holotype habitus, dorsal view, body length 6.5 mm.

Figs 1 [Fig F2] [Fig F3] [Fig F4] 5

#### Type material.

**Holotype** female, EGYPT: Alexandria: Borg El-Arab, 30.8856°[N], 29.5834°[E], 11.viii.1934, Shafik.

The type specimen is mounted with minuten pin on circular card, with two rectangular labels: Burg 11.7.34 [handwritten, black ink] / Zool. Dep. Collection, Egyptian University, Collector [printed, black ink, all caps] Shafik [handwritten, black ink] (Cairo University Collection (CUC)).

#### Diagnosis.

See genus diagnosis.

#### Description.

Body Length= 6.5 mm (female). *Head*. Head very wide, 2x head length (excluding antennae); eyes widely separated, frons width at narrowest point 3x width of ocellar tubercle; eye relatively small and globose; frons broadly concave, glossy brown with glaucous (pale bluish) grey pubescence, irregular raised callosities around antennal base and along eye lower margin, callosities with dark erect setae of varying length; parafacial with silver pubescence and glabrous band between antennal base and eye margin; occiput broadly rounded convex with glaucous grey pubescence and scattered erect black macrosetae, longer macrosetae along postocular ridge, medium length on occiput admixed with very short setae; gena rounded with grey-silver pubescence, admixed with short, erect white setae, darker anteriorly, angular process projecting anteriorly from gena with short dark setae; mouthparts reduced, proboscis narrow, barely protruding from oral cavity, palpus not observed as oral cavity obscured; antenna 2× length of head; scape shorter than head length, glossy dark yellow, bulbous and non-symmetrical in lateral view, with dark erect macrosetae (longer ventrally), absent medially, sparse pubescence on medial surface; pedicel brown with row of erect dark macrosetae; flagellum length equal to head, basal flagellomere large, paddle-shaped, style terminal. *Thorax*. Cuticle dark yellow to brown with dense glaucous grey pubescence, scutum with diffuse medial stripe and light brown patches laterally on prescutal area and supra-alar callus; scutal pile as short dark setae; scutellum dark yellow, overlain with uniform grey pubescence; pleuron dense glaucous grey pubescent with short pale setae on postpronotum, postcervical sclerite, anepisternum, and katatergite; coxae grey glaucous pubescent with short pale setae on anterior surfaces, macrosetae few in number, black; legs dark brown with short dark setae; basitarsus equal length to remaining tarsomeres combined; tarsal segments with short macrosetae apically; tarsal claws short, straight, elongate terminal setae extending from apical segment (one setae on fore and mid leg, three setae on hind leg); haltere stem light brown, knob cream; wing membrane smoky infuscate due to dense microtrichia, darker anteriorly around pterostigma; scutal chaetotaxy (macrosetae pairs): notopleural, 3; supra alar, 1; post alar, 1; dorsocentral, 2; scutellar, 1. *Abdomen*. Cuticle glossy dark brown, posterior margins and intersegmental membranes of all segment yellow to white, tergites dark yellow along narrow posterolaterally margin; sparse short dark setae on all segments, denser posteriorly and on terminalia. *Female genitalia*. Acanthophorite spines reduced in size; sternite 8 emarginate along posterior margin, upturned and strongly convex with elongate setae admixed with shorter setae; furca rectangular, broad at base with two openings.

**Figure 2. F2:**
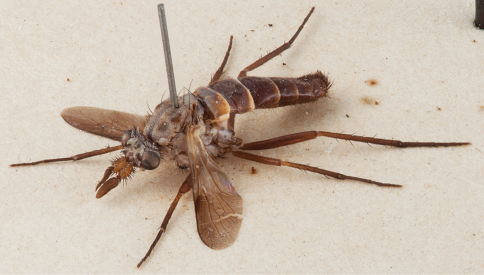
*Salwaea burgensis* gen. et sp. n., female Holotype habitus, oblique view, body length 6.5 mm.

#### Etymology.

The species epithet is derived from the type locality, Burg (meaning tower in Arabic), or more recently Borg El-Arab, and is located within Alexandria, Egypt.

#### Comments.

The male of *Salwaea burgensis* gen. *et* sp. n. is unknown. The body length is relatively short with a body length of 6.5 mm in the only known female specimen. The type locality is today very close to developed urban areas and this species may possibly be locally extinct there. It is remarkable that despite modern collecting efforts in Egypt and adjacent countries, no further specimens of this stiletto fly have been found.

**Figure 3. F3:**
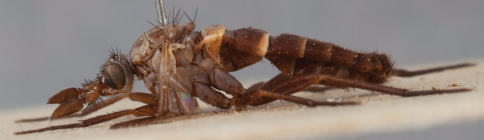
*Salwaea burgensis* gen. et sp. n., female Holotype habitus, lateral view, body length 6.5 mm.

**Figure 4. F4:**
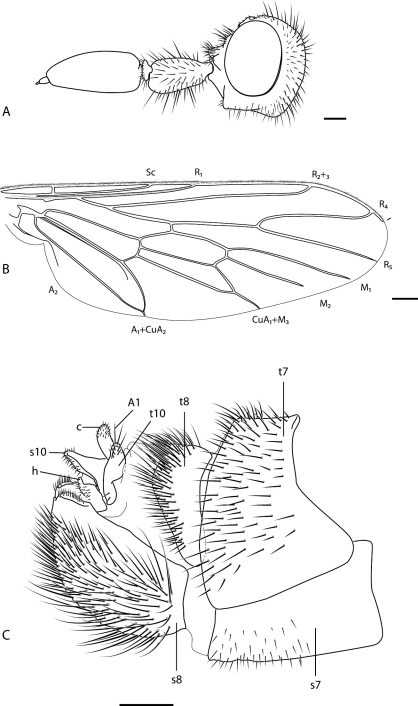
*Salwaea burgensis* gen. et sp. n. **A** female head, lateral view **B** wing **C** female terminalia, lateral view. Abbreviations: **A1** acanthophorite spines **c** cercus **h** hypoproct **t7–10** tergites 7–10 **s7–10** sternites 7–10. Scale line = 0.2 mm.

**Figure 5.**
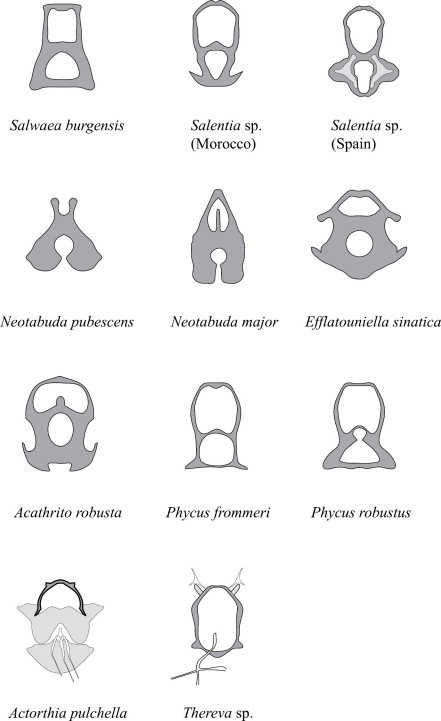
*Salwaea burgensis* gen. et sp. n., Female furca of various Phycinae. *Thereva* included for comparison. Figures not to scale.

### Key to Palaearctic genera of Phycinae

**Table:** 

1	Wing costal vein ends in radial or medial fields	2
–	Wing costal vein ends at CuA_2_+A_1_	6
2	Flagellum large and paddle-shaped (Fig. 4A); costal vein ending just past R_4_ (Figs 1, 4B)	*Salwaea* gen. n
–	Flagellum turbinate, conical to elongate cylindrical, never paddle-like; costal vein terminating at or beyond R_5_	3
3	Wing costal vein ends at CuA_1_+M_3_; male frons wider than ocellar tubercle	*Yemenia* Koçak & Kemal, 2009
–	Wing costal vein ends at R_5_, M_1_ or M_2_; male with eyes contiguous	4
4	Head height greater than length; frons usually with black spot or line medially; costal vein ends at R_5_; relatively small individuals (< 5.0 mm body length)	*Efflatouniella* Kröber, 1927 (in part)
–	Head height subequal to length; frons usually without medial mark; costal vein ends in medial field; larger individuals, usually greater than 5.0 mm	5
5	Wing costal vein ending at M_2_	*Ruppellia* Wiedemann, 1830
–	Wing costal vein ending at M_1_	*Acathrito* Lyneborg, 1983
6	Flagellum elongate, longer than head	*Phycus* Walker, 1850
–	Flagellum shorter than head	7
7	Hind coxal knob absent	*Actorthia* Kröber, 1912 (13 spp.)
–	Hind coxal knob present	8
8	Head height greater than length; frons usually with black spot or line medially; body covered with extensive glaucous grey pubescence; prosternum without pile in central depression; wing with discal cell truncated basally; antennal scape not elongate or bulbous; small specimens (body length < 5.0 mm body length)	*Efflatouniella* Kröber, 1927 (in part).
–	Head height subequal to length; frons usually without medial mark; body uniformly black, without extensive grey pubescence; prosternum with pile in central depression; wing with discal cell acute basally; antennal scape elongate, frequently bulbous; larger specimens (body length usually > 5.0 mm)	*Salentia* Costa, 1857

## Supplementary Material

XML Treatment for Salwaea

XML Treatment for Salwaea burgensis
